# Contralateral Anterior Interhemispheric–Transcallosal–Transrostral Approach for the Resection of a Subcallosal Cavernous Malformation: A Case Report and an Operative Video

**DOI:** 10.3389/fsurg.2022.902242

**Published:** 2022-06-10

**Authors:** Aderaldo Costa Alves, Marco Antônio Zanini, Pedro Tadao Hamamoto Filho, Feres Eduardo Aparecido Chaddad-Neto

**Affiliations:** ^1^ Sunnybrook Health Sciences Centre, University of Toronto, Toronto, ON, Canada; ^2^ Departamento de Neurologia, Psicologia e Psiquiatria, Universidade Estadual Paulista, Botucatu, Brazil; ^3^ Departamento de Neurologia e Neurocirurgia, Universidade Federal de São Paulo, São Paulo, Brazil

**Keywords:** Interhemispheric approach, cerebral cavernous malformation (CCM), brain surgery, microsurgery, lateral decubitus position

## Abstract

This case report demonstrates the surgical resection of a cerebral cavernous malformation located in the subcallosal region. The authors present a detailed operative video explaining the steps to successfully remove the lesion through a contralateral interhemispheric–transcallosal–transrostral approach with the patient in lateral decubitus. The surgical procedure was uneventful, and the patient had no postoperative deficits and no residual lesions in a three-month follow-up.

## Introduction

The resection of lesions situated in the subcallosal region is challenging for neurosurgeons given the deep location adjacent to important structures that could potentially cause severe neurological impairment if damaged, not to mention the high surgical complexity to access this area. The standard described approaches to the subcallosal region include the trans-sylvian approach with a fronto-orbitozygomatic craniotomy, subfrontal interhemispheric (anterior route), and a transventricular preforniceal approach (1–3). All these approaches require the dissection and/or retraction of critical structures, so individual anatomical aspects of each case must be considered during surgical planning.

One of the key principles for the resection of cerebral cavernous malformation (CCM) surgery is to approach the lesion through its most superficial surface to minimize the dissection and damage of normal tissue. This is especially important when the CCM is located in highly eloquent areas.

We report a case of a patient who underwent surgical resection of a CCM situated in the subcallosal area *via* an interhemispheric–transcallosal–transrostral approach. Different from the usual technique, described through the same side of the lesion and in the supine position, we performed a contralateral approach with the patient in a lateral decubitus to reach the cavernoma with a straight surgical view and minimal brain retraction. A detailed surgical video illustrates and explains the nuances of the surgical procedure and the advantages of unconventional positioning in this approach.

## Case Description

A healthy 49-year-old female presents with a recent worsening of chronic headaches. A brain MRI was done, revealing a moderate size CCM located in the right subcallosal region. Management options were deeply discussed with the patient. Conservative management was recommended given the low risk of hemorrhage in supratentorial cavernomas; however, the patient was not comfortable living with any risks of brain hemorrhage and opted for surgical resection. Informed consent was obtained.

During the surgical planning, considering that the cavernoma was superficial on the floor of the right frontal horn, an interhemispheric–transcallosal–transrostral approach was chosen. To access this area with minimal brain retraction, we adapted the usual technique by positioning the patient in a left lateral decubitus, so the gravitational force would naturally shift the deep central brain structures downward, while the falx cerebri would hold the right hemisphere in place, allowing for a contralateral approach from the left side to reach the right frontal horn.

### Surgical Description

**Figure 1:**
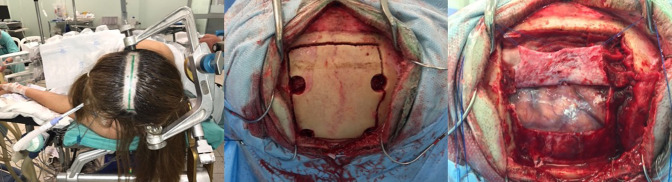
Left: Patient positioned in a left lateral decubitus, with the incision marked. Middle*:* Intraoperative view after a left frontal-parietal craniotomy crossing the midline was performed. Right*:* Surgical view after dura mater opening.

**Figure 2:**
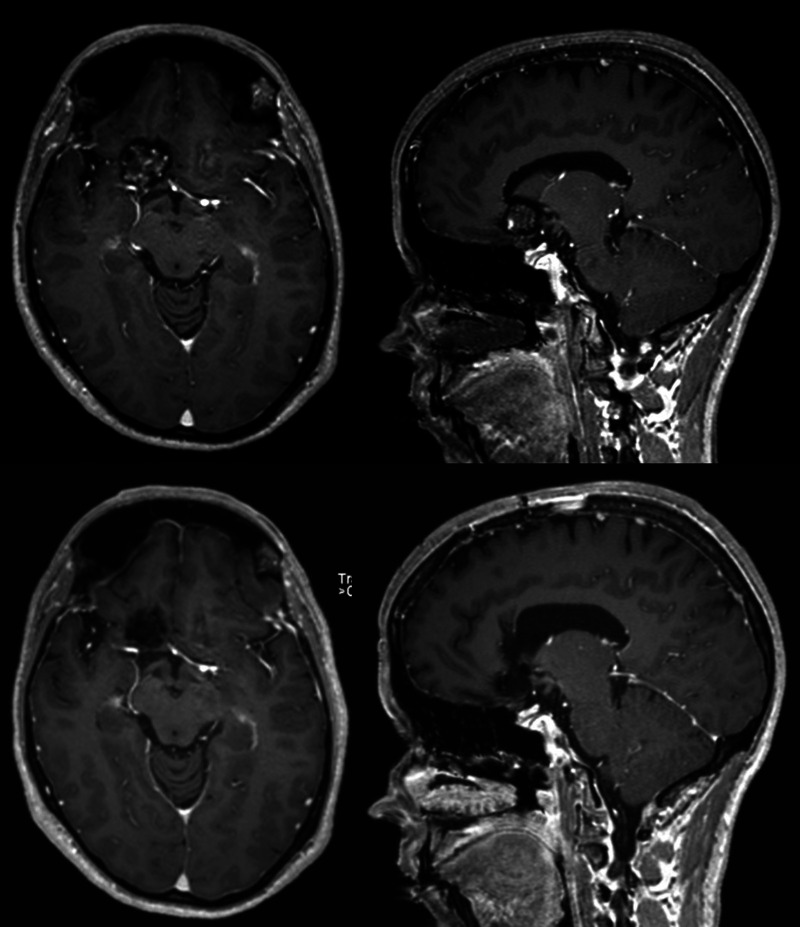
Brain MRI T1-post-gadolinium comparison between before (top) and after (bottom) surgical resection of a right subcallosal cavernoma.

The patient was positioned in a left lateral decubitus, with the head fixed by a three-point fixation system. A left frontal–parietal craniotomy crossing the midline was performed. The dura mater was opened on the left side in a “C” shape, with the base on the sagittal sinus. The left cerebral hemisphere was exposed, while the right hemisphere was held in position by the falx. The left interhemispheric dissection was done until the visualization and opening of the corpus callosum. The callosotomy led straight to the right frontal horn of the lateral ventricle. Neuronavigation was also used to guide surgical localization. An area with hemosiderin deposition was visualized on the floor of the right frontal horn, indicating the location of the CCM. Once the lesion was localized, a microsurgical technique was used to completely resect it. Hemostasis was achieved, and standard closure was performed (refer to the surgical Supplementary Video and Figures 1, 2).

Postoperatively, the patient had no neurological deficits and was discharged home in good condition. A three-month follow-up MRI confirmed the total resection. She also continued to have no neurological symptoms during follow-up one year later.

## Discussion

Lesions located in the subcallosal region are usually resected *via* different approaches that carry a considerable risk of neurological damage.

The trans-sylvian approach with a fronto-orbitozygomatic craniotomy is the inferior route, ideal for lesions located adjacent to the anterior circulation arteries. In such cases, it gives access with lesser brain tissue removal in comparison to other approaches due to the fact that most of the dissection is in the subarachnoid space. However, to reach the lesions, it requires the manipulation of crucial perforating vessels arising from the internal carotid and anterior cerebral arteries, imposing risks of infarction and potentially causing significant deficits (1).

The anterior route can be done with a subfrontal interhemispheric approach, commonly used for anterior communicating aneurysms or midline suprasellar tumors. To access the subcallosal area through this approach, there are also risks of damage to anterior cerebral and anterior communicating perforating branches, associated with deficits such as memory impairment, amnestic-confabulatory syndrome, or hypothalamic dysfunction, in case of injury to subcallosal or hypothalamic branches (4).

The transcortical–transventricular–preforniceal approach is one of the possible superior routes, usually chosen for intraventricular lesions but also feasible to access the subcallosal region. This approach avoids manipulation of perforating arteries but requires a significant amount of brain transgression and is associated with potential injury to the fornix, leading to memory impairment (1–3).

In this case, we present the successful resection of a subcallosal cavernous malformation and explain the steps involved in this approach through a contralateral interhemispheric–transcallosal–transrostral route. A contralateral interhemispheric approach to deep-seated cavernous malformations allows for a great exposure (5), and a transrostral opening provides access to the subcallosal region.

This approach has been previously described in a cadaveric study (4). Its main advantage is to avoid manipulation of perforating vessels or structures such as the fornix. Another benefit of this alternative technique is the straight corridor to the subcallosal area provided by the brain shift due to gravitational force when the patient is positioned in a lateral decubitus. However, a callosotomy, as needed in this approach, is known to potentially cause neurological impairment, including memory deficits, disturbances in interhemispheric transfer of learning, and the cognitive components of executive functions (6). Therefore, the minimum opening of the corpus callosum should be aimed.

In conclusion, the contralateral interhemispheric–transcallosal–transrostral approach in lateral decubitus seems to be a relatively safe alternative for lesions located in the subcallosal region.

## Data Availability

The original contributions presented in the study are included in the article/Supplementary Material; further inquiries can be directed to the corresponding author/s.
